# Effects of transcutaneous electric acupoint stimulation on drug use and responses to cue-induced craving: a pilot study

**DOI:** 10.1186/1749-8546-7-14

**Published:** 2012-06-10

**Authors:** David M Penetar, Anthony Burgos-Robles, George H Trksak, Robert R MacLean, Steven Dunlap, David Y-W Lee, Scott E Lukas

**Affiliations:** 1 Behavioral Psychopharmacology Research Laboratory, McLean Hospital, 115 Mill Street, Belmont, MA, 02478, USA; 2 Department of Psychiatry, Harvard Medical School, 115 Mill Street, Belmont, MA, 02478, USA; 3 Bio-Organic and Natural Products Laboratory, McLean Hospital, 115 Mill Street, Belmont, MA, 02478, USA; 4 McLean Hospital/Harvard Medical School, 115 Mill Street, Belmont, MA, 02478, USA

## Abstract

**Background:**

Transcutaneous electric acupoint stimulation (TEAS) avoids the use of needles, and instead delivers a mild electric current at traditional acupoints. This technique has been used for treating heroin addiction, but has not been systematically tested for other drugs of abuse. This study aims to investigate the effects of TEAS on drug addiction.

**Methods:**

Volunteers who were either cocaine-dependent (n = 9) or cannabis-dependent (n = 11) but were not seeking treatment for their dependence participated in a within-subject, single-blind study. Treatment consisted of twice daily 30-minute sessions of TEAS or sham stimulation for 3.5 days. The active TEAS levels were individually adjusted to produce a distinct twitching response in the fingers, while the sham stimulation involved 2 minutes of stimulation at threshold levels followed by 28 minutes of stimulation below the detection levels. The participants recorded their drug use and drug cravings daily. At 1 hour after the last morning session of TEAS or sham stimulation, a cue-induced craving EEG evaluation was conducted. Event-related P300 potentials (ERPs) were recorded, sorted, and analyzed for specific image types (neutral objects, non-drug-related arousing images, or drug-related images).

**Results:**

TEAS treatment did not significantly reduce the drug use or drug cravings, or significantly alter the ERP peak voltage or latency to peak response. However, the TEAS treatment did significantly modulate several self-reported measures of mood and anxiety.

**Conclusion:**

The results of this pilot study with a limited sample size suggest that the acupoint stimulation techniques and protocol used in this trial alone do not significantly reduce cravings for or use of cocaine or cannabis. The findings that TEAS modulates mood and anxiety suggest that TEAS could be used as an adjunct in a multimodal therapy program to treat cocaine and cannabis dependence if confirmed in a full randomized controlled clinical trial.

## Background

Drug abuse and dependence are medical problems that have impacts on the personal, social, and economic fabric of society. Searches for effective therapies have been, and continue to be, important areas of research in neuroscience and pharmacology, as these disciplines seek to develop a greater understanding of the biological basis of drug abuse. Although there are several approved interventions for treating tobacco, alcohol, and opioid addiction, there remains a lack of effective medical treatments for cocaine and cannabis abuse.

Traditional acupuncture methods involving the insertion of needles at specific points on the body have been used for centuries to treat a variety of maladies [1]. A review of controlled clinical trials by the World Health Organization has documented that drug dependence (specifically alcohol, cocaine, opiates/heroin, and tobacco dependence) is a condition for which acupuncture may have therapeutic benefits, but additional studies are warranted [2]. Owing to the widespread use of acupuncture techniques to treat abuse problems related to alcohol, nicotine, and opiates, numerous researchers have attempted to systematically assess its usefulness in treating cocaine abuse. Most commonly, auricular acupuncture techniques (involving needle insertion at specific locations on the outer ear) have been tested with mixed results [3-6]. Although a well-controlled study on methadone-maintained cocaine-dependent subjects showed a significant decrease in cocaine-positive urine samples after 8 weeks of treatment [4], a larger-scale study by the same research group concluded that acupuncture was no better than a needle-insertion control condition or a relaxation control condition in these dually-dependent subjects [7]. Two systematic reviews of the available literature concluded that the currently available acupuncture techniques are not effective in treating cocaine abuse [8,9].

Alternative strategies involve application of electrical stimulation to needles inserted into traditional acupuncture points or simply application of electrical stimulation *via* electroconductive pads on the skin at these acupoints. These techniques have been found to be effective in reducing the severity of heroin withdrawal symptoms [10] and relapse [11]. Evidence for the possible effectiveness of these techniques for cocaine dependence may be inferred from an animal study, which showed that cocaine-induced conditioned place preference in rats was blocked by application of electroacupuncture at 100 Hz, but not at 2 Hz [12]. Neither traditional needle insertion nor electrical stimulation has been assessed in cocaine- and cannabis-dependent individuals.

Transcutaneous electric acupoint stimulation (TEAS) has been shown to promote the release of endorphins [1], which may be the basis of its effectiveness for opiate addiction, and also lead to the release of a variety of neuropeptides that control important brain functions [13]. This study aims to explore the feasibility of TEAS as a possible means for reducing drug use and drug cravings in cocaine- and cannabis-dependent subjects.

## Methods

### Participants

Cocaine- and cannabis-dependent individuals who were not seeking treatment for their dependence were recruited by advertisements in local newspapers and on the internet. The protocol and informed consent forms were reviewed and approved by the McLean Hospital Institutional Review Board. The participants were briefed regarding the purpose and requirements of the study, and then given time to read the informed consent form in detail. After an opportunity to ask questions and ensure understanding, written informed consent was obtained. The participants underwent a physical examination by a physician and a mental health examination, including a Structured Clinical Interview according to DSM-IV criteria (SCID) [14], under the supervision of a clinical psychologist. The inclusion criteria for participation consisted of being physically healthy, having no Axis I psychological disorder (*i.e.*, serious mental disorder such as major depression, bipolar disorder, or schizophrenia), and meeting dependence criteria for either cocaine or cannabis. Dependence was assessed during the SCID by evaluating whether a maladaptive pattern of substance use was occurring. Specifically, the interviewer evaluated each participant for tolerance, withdrawal, loss of control, preoccupation with obtaining and using the drug, impacts on social, occupational, or recreational activities, and use despite physical harm. Individuals were excluded from participation if they had abnormal blood and urine chemistry values, had an abnormal electrocardiogram, were taking prescription medications, or had a history of head trauma or major head injury. Four individuals did not qualify after the physical examination and mental health assessments, and two were excluded from the data analysis because they did not report any drug use during the baseline period. Of the remaining 20 participants who completed the study (11 men, 9 women; mean age, 33.2 ± 9.2 years; age range, 21 – 49 years), nine met the criteria for cocaine dependence (three crack cocaine users) and 11 met the criteria for cannabis dependence. The individuals were paid for their participation.

### Equipments

**Figure 1:**
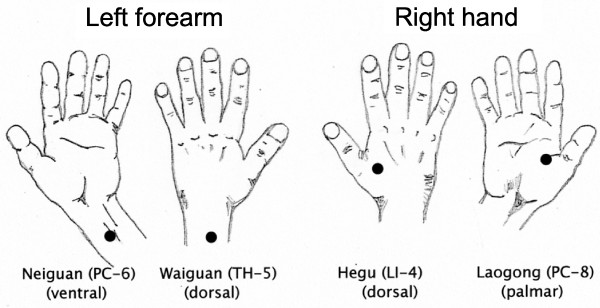
**Acupoints for electrical stimulation with the HANS device were located by visual inspection, measurement, and palpation of the participant’s hands and forearm/wrist.** Four acupoints were stimulated: PC-6 (*Neiguan*), 2 inches (*i.e.* 2 *cun*) proximal of the palm, between the palmaris longus tendon and flexor carpi radialis tendon; TH-5 (*Waiguan*), 2 inches (*i.e.* 2 *cun*) proximal of the skin crease on the back of the wrist, between the ulnar and radius bones; LI-4 (*Hegu*), at the midpoint of the second metacarpal bone on the radial side; and PC-8 (*Laogong*), midway between the second and third metacarpal on the palmar side.

TEAS was delivered by a Han’s Acupoint Nerve Stimulator (HANS) (Hans International Inc., China). This battery-operated device delivered electrical stimulation *via* two pairs of outputs to electroconductive pads placed at acupoints used in previous studies of heroin addiction and treatment of withdrawal [10,11,15]. Dr. Ji-Sheng Han, the developer of the HANS device, personally trained the research staff to use the device and provided advice for the frequencies, durations, and placements to be used based on his extensive work in the field [12,16,17]. Acupoints are shown in Figure 1 and are described in more detail below. The stimulation comprised alternating 3-second intervals of pulses at frequencies of 2 and 100 Hz using intensities that were sufficiently strong to elicit a slight, but noticeable, twitch in the index finger or thumb of each hand. The mean intensity for the PC-6/TH-5 acupoint was 9.5 ± 1.7 mA (range, 5.7 – 12.7 mA), while that for the LI-4/PC-8 acupoint was 12.6 ± 3.0 mA (range, 7.7 – 18.1 mA). For the sham stimulation, the participants received stimulation at detectable threshold levels (approximately one-half of the ‘twitch’ levels) for 2 minutes, followed by stimulation at the undetectable level of 1 mA for the remaining 28 minutes of the session. The delivered current levels were concealed from the participants.

Brain electroencephalographic (EEG) recordings of evoked potentials were acquired using an Electro-Cap (Electro-Cap International, USA) with 32 tin scalp electrodes and two electro-oculographic electrodes. The scalp electrodes were positioned in accordance with the 10–20 convention [18], affixed to the scalp with Electro-Gel (Electro-Cap International), and referenced to a nose electrode to create reference-free images. The interelectrode impedances were < 10 kOhms at 30 Hz with online wide bandpass filtering of 0.001 – 100 Hz and a gain of 20,000 (EPA-6 Electrophysiology Amplifiers; Sensorium Inc., USA).

### Procedures

The study was a single-blind, sham-controlled, crossover design. The participants were told that the study was being conducted to assess the effects of different levels of electrical stimulation on drug use and drug cravings, and that they may or may not feel the stimulation.

#### Treatment

Following a baseline week during which the participants recorded their drug use and drug cravings in daily diaries, two 3.5-day treatment periods were conducted with a 2-week ‘wash-out’ period between the two periods (Table 1). In the diaries, the participants indicated their drug use and rated their drug cravings on a scale of 0 (none at all) to 10 (extreme). In addition, they rated themselves on seven items, comprising mood, concentration, appetite, sleep quality, anxiety, irritability, and tension/agitation, using a 100-mm visual analog scale. The order of the TEAS and sham stimulation treatments was counterbalanced among the participants. The treatment periods consisted of two 30-minute sessions of stimulation (separated by 3 to 6 hours) per day for 3 consecutive days. On day 4, the participants came to the laboratory for a final 30-minute session of stimulation, followed by a cue-induced craving EEG evaluation after 1 hour.

**Table 1:** Summary of procedure. Treatment periods were either with sham stimulation or active current presented in a counterbalanced order across subjects

**Study phase**	**Events**	**Notes**
Baseline period	Daily diaries for drug use and cravings	Recorded for 1 week
Treatment 1	Two 30-minute sessions for 3 1/2 days	Sessions separated by 3 to 6 hours
Cue-induced craving EEG session	Event-Related Potential (ERP) recordings following neutral and drug-related cues	Session began 1 hour after last Transcutaneous Electric Acupoint Stimulation (TEAS) treatment
Washout period	2 weeks in length	No treatments; continue to record drug use and cravings in diaries
Treatment 2	Two 30-minute sessions for 3 1/2 days	Sessions separated by 3 to 6 hours
Cue-induced craving EEG session	Event-Related Potential (ERP) recordings following neutral and drug-related cues	Session began 1 hour after last Transcutaneous Electric Acupoint Stimulation (TEAS) treatment

#### Electroconductive pad placement

At each treatment session, the four specific acupoints for placement of the electroconductive pads of the HANS stimulation device were located by visual inspection, measurements, and palpation of the participant’s hands and forearms/wrists. The targeted acupoints were as follows (Figure 1): PC-6 (*Neiguan*), 2 inches (*i.e.* 2 *cun*) proximal to the palm between the palmaris longus tendon and flexor carpi radialis tendon; TH-5 (*Waiguan*), 2 inches (*i.e.* 2 *cun*) proximal to the skin crease on the back of the wrist between the ulna and radius bones; LI-4 (*Hegu*), at the midpoint of the second metacarpal bone on the radial side; and PC-8 (*Laogong*), midway between the second and third metacarpals on the palmar side.

#### EEG recordings

The cue-induced craving EEG evaluation investigated brain event-related potentials (ERPs) and specifically probed the production of the P300 wave response while the participants viewed photographs of objects from three categories: neutral (furniture such as chairs, lamps, baskets); non-drug-related arousing (shark attack, pointed gun, ferocious barking dog); and drug-related. The drug-related photographs were specific to the participant’s drug of choice (scenes of drug paraphernalia and drug use). An oddball visual procedure presented the images in a pseudorandom order using the following proportions: 60% common/frequent neutral pictures and 40% target/rare pictures (20% drug-related and 20% arousing). Two successive rare images were never presented. The images were displayed for 750 ms at 1-second intervals. The participants were asked to differentiate between drug scenes and non-drug scenes (neutral or arousing) by pressing a corresponding button on a computer mouse. The first EEG/ERP session was conducted after a short acclimation period in the recording chamber. A second EEG/ERP session was conducted 26 minutes later, after the participants had handled neutral objects such as common office items (*e.g.*, pens, paper, staplers) for 7 minutes. A third EEG/ERP session was conducted 26 minutes later, after the participants had handled drug-related objects (*e.g.*, drug paraphernalia). The cocaine-dependent participants were allowed to handle paraphernalia associated with smoked (crack) or snorted cocaine. The cannabis-dependent participants were allowed to handle and smell a used pipe that contained residual byproducts of burned marijuana. At four times during the ERP sessions, the participants answered two questions of “Would you use cocaine (or marijuana) now if you could?” (Yes/No) and “What is your desire to use cocaine (or marijuana) right now?”. The responses to the latter question were evaluated using a 100-mm visual analog scale from ‘none at all’ to ‘extreme’. Regarding the timing, the questions were asked before and after the participants handled the neutral objects before the second EEG/ERP session, and before and after the participants handled the drug-related objects before the third EEG/ERP session.

### Data analysis

ERPs were analyzed using Brain Vision Analyzer version 1.05.0004 (Brain Products GmbH, Germany). The processing included screening for artifacts, filtering (Butterworth zero phase, cutoff = 40 Hz, slope = 48 dB/oct), and ocular correction, 10 automated artifact rejections, and baseline correction. The trials for each picture type (neutral, arousing, or drug-related) were averaged, and the amplitude and latency to peak response of the P300 waveforms were identified for each participant based on the largest positive peak between 300 and 600 ms. The waveforms and P300 amplitudes showed no differences between the left and right electrodes. Each participant’s drug craving and drug use data were averaged across days within each study phase before combination across subjects.

The data were presented as the mean ± standard deviation (SD). Statistical analyses for all dependent measures were conducted with SPSS 13.0 for Mac OS X (SPSS Inc., USA) using a linear mixed-effects model repeated-measures one-way analysis of variance (ANOVA). When appropriate, *post hoc* analyses of pair-wise comparisons were conducted with Fisher’s least significant difference test. Statistical significance for all tests was set at *P* < 0.05.

## Results

### Drug cravings and drug use during treatment

**Figure 2:**
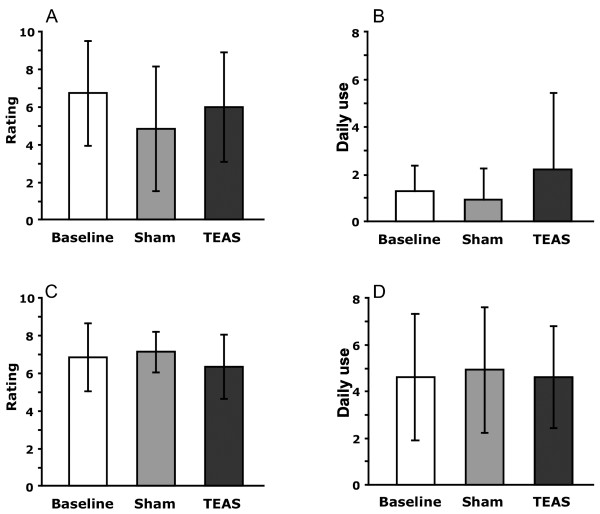
**Craving scores and daily use mean ± SD by phase of the study for cocaine and marijuana participants.** Cravings were ratings on a 10-point scale from 1-‘none’ to 10-‘extremely’. ‘Baseline’ refers to assessments during the one week before any treatment was conducted. **A**: Cocaine craving; **B**: Cocaine use; **C**: Marijuana craving; **D**: Marijuana use.

The participants provided daily overall ratings of their cravings for cocaine or cannabis during the previous 24-hour period, and self-reported their daily use of cocaine or cannabis (Figure 2, Table 2). The self-reported cravings averaged between 6 and 7 on a 10-point scale during the baseline week before the treatments began. These ratings did not change significantly during treatment with either the TEAS or sham stimulation of the acupoints. The cocaine-dependent participants reported using cocaine twice a day on average, while the cannabis-dependent participants used cannabis four times a day on average. These rates of use were not significantly altered by either treatment.

**Table 2:** Drug use and cravings during phases of the study (mean ± SD)

	**Mean ± SD**	**95% confidence interval**
Cocaine use		
Baseline	1.22 ± 1.1	[−0.34, 2.8]
Sham	0.9 ± 1.3	[−0.58, 2.4]
TEAS	2.17 ± 3.2	[0.69, 3.6]
Cocaine cravings		
Baseline	6.7 ± 2.8	[4.5, 8.9]
Sham	4.8 ± 3.3	[2.7, 6.9]
TEAS	5.96 ± 2.9	[3.8, 8.0]
Marijuana use		
Baseline	4.6 ± 2.7	[2.7, 6.4]
Sham	4.9 ± 2.7	[3.4, 6.5]
TEAS	4.6 ± 2.2	[3.1, 6.2]
Marijuana cravings		
Baseline	6.8 ± 1.8	[5.7, 7.9]
Sham	7.1 ± 1.1	[6.2, 8.2]
TEAS	6.3 ± 1.7	[5.4, 7.4]

### Mood measurements during treatment

**Figure 3:**
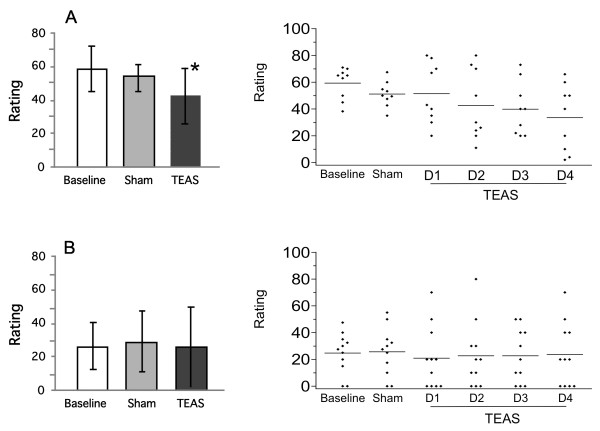
**Ratings of anxiety during each phase of the study for cocaine and marijuana participants.** Anxiety was rated on a 100 mm line with anchors of ‘none’ to ‘extremely’. Left panels are group mean ± SD; right panels are individual participant scores (averaged during Baseline and Sham, but by separate days during TEAS treatment).* significantly different from baseline, *P* < 0.05. **A**: Anxiety of cocaine subjects; **B**: Anxiety of marijuana subjects.

The self-reported ratings for anxiety in both sets of participants are shown in Figure 3. Overall, the cocaine-dependent participants had higher ratings for anxiety than the cannabis-dependent participants in all phases of the study. TEAS treatment significantly reduced the anxiety ratings in the cocaine-dependent participants (F(2,16) = 5.44, *P* = 0.016). The daily ratings declined over the treatment days (Figure 3, top right panel). *Post hoc* analyses revealed that the mean ratings on day 4 of TEAS treatment were significantly lower than those on the baseline days. The ratings for anxiety in the cannabis-dependent participants did not change significantly during the treatment. Several additional measures were significantly altered during the treatment (Table 3). TEAS significantly reduced tension/agitation and irritability, and improved concentration in the cocaine-dependent participants. Similarly, significant decreases in tension/agitation and irritability were found in the cannabis-dependent participants.

**Table 3:** Ratings on 100 mm visual analog scales during phases of study (mean ± SD)

	**Baseline**	**Sham**	**TEAS**	
**Cocaine**				
Anxiety	58.9 ± 12.7	53.2 ± 7.6	42.4 ± 16.1*	*P* = .007
Mood	44.4 ± 24.9	60.5 ± 20.4	56.2 ± 21	
Concentration	45.7 ± 22.4	60 ± 22.1	59.6 ± 17.6*	*P* = .045
Appetite	44.3 ± 19.5	53.3 ± 13.2	54.8 ± 21	
Sleep quality	50.8 ± 25.5	59.2 ± 19.8	54.9 ± 28.3	
Irritability	53.7 ± 16.5	46.4 ± 7.2	34.6 ± 21.6*	*P* = .047
Tension & agitation	50.1 ± 17.7	32.8 ± 17.5	23.7 ± 17.2*	*P* = .0004
**Marijuana**				
Anxiety	26.3 ± 14.2	29.1 ± 18.4	25.9 ± 23.5	
Mood	48.8 ± 14.9	55.2 ± 18.4	54.1 ± 21.2	
Concentration	54.2 ± 20.8	55.5 ± 24.1	59.8 ± 22.8	
Appetite	43.7 ± 16.2	45.2 ± 22.8	49.5 ± 17.8	
Sleep quality	69.3 ± 14.9	63.6 ± 22.1	64.3 ± 21.1	
Irritability	32.0 ± 18.8	29.1 ± 18.2	22.3 ± 17.8*	*P* = .05
Tension & agitation	26.5 ± 21.1	23.2 ± 19.1	12.0 ± 13.2*	*P* = .05

*Significantly different from Baseline.

### EEG findings

Analyses of the four central recording electrodes (Oz, Pz, Cz, and Fz) were conducted in all participants. At each electrode, the peak response voltage and latency to peak response of the P300 waveform were analyzed.

**Figure 4:**
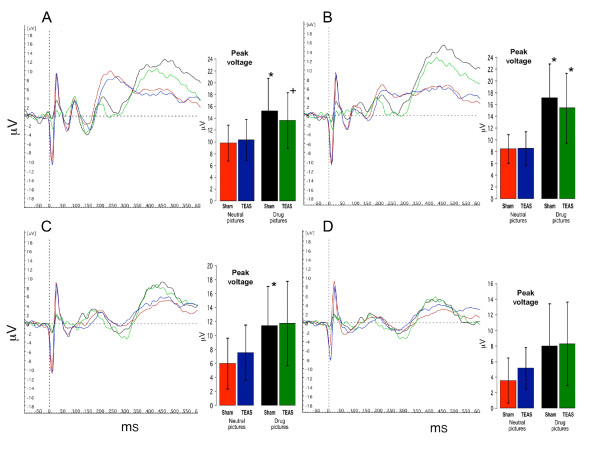
**Averaged ERP for four conditions in cocaine abusing subjects (n = 9).** Red lines and bars represent averaged responses to neutral pictures after sham TEAS treatment; Blue lines and bars represent responses to neutral pictures after active TEAS treatment; Black lines and bars represent responses to drug pictures after sham TEAS treatment; Green lines and bars represent responses to drug pictures after active TEAS treatment. Bar graph inserts show magnitude of peak response in μV (mean ± SD) averaged across subjects. * indicates significant differences between responses to drug pictures and neutral pictures (*P* < 0.05). + indicates a statistical trend (*P* = 0.06). **A**: Oz; **B**: Pz; **C**: Cz; **D**: Fz.

**Figure 5:**
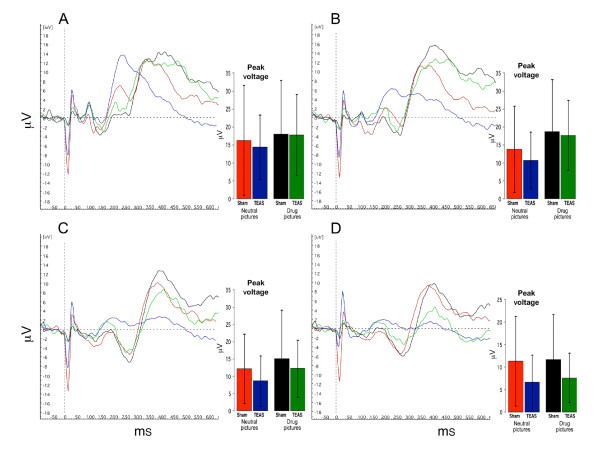
**Averaged ERP for four conditions in marijuana abusing subjects (n = 11).** Red lines and bars represent averaged responses to neutral pictures after sham TEAS treatment; Blue lines and bars represent responses to neutral pictures after active TEAS treatment; Black lines and bars represent responses to drug pictures after sham TEAS treatment; Green lines and bars represent responses to drug pictures after active TEAS treatment. Bar graph inserts show magnitude of peak response in μV (mean ± SD) averaged across subjects. **A**: Oz; **B**: Pz; **C**: Cz; **D**: Fz.

Figures 4 and 5 show the summed ERP responses for the four specific conditions of the study, comprising the ERPs for viewing neutral and drug-related pictures according to the treatment condition. These data were collected during the third ERP session, in which the participants handled drug-related paraphernalia before viewing the pictures. The mean peak voltage values for each condition are shown in the bar graphs accompanying the ERP lines (values are presented in the Additional file 1: Supplemental tables). For the cocaine-dependent participants (Figure 4), there was a main effect for picture type, and the responses to the neutral pictures were lower than the responses to the drug pictures for Oz (F(5,40) = 2.749, *P* = 0.032), Pz (F(5,40) = 7.105, *P* < 0.001), and Cz (F(5,40) = 2.809, *P* = 0.029). The responses to the drug-related pictures recorded at Fz did not differ significantly from the responses to the neutral pictures. There was no effect of TEAS treatment, since the responses to the drug-related pictures and neutral pictures did not differ significantly following the TEAS and sham stimulation treatments. For the cannabis-dependent participants (Figure 5), the responses did not differ significantly by treatment or picture type. In both groups of participants, *post hoc* analyses revealed that the responses to the non-drug-related arousing pictures differed significantly from those to the neutral pictures, but did not differ significantly from the responses to the drug-related pictures (data not shown).

The latencies to peak responses were not altered by the treatment or picture type for three of the center line electrodes, Pz, Cz, and Fz (values presented in the Additional file 1: Supplemental tables). For Oz, however, the latency to peak response was longer for the drug-related pictures than for the neutral pictures in both the cocaine-dependent (F(5,40) = 6.797, *P* < 0.001) and cannabis-dependent (F(5,50) = 6.104, *P* < 0.001) participants. In a similar manner to the peak response results, *post hoc* analyses revealed that the latency measures for the ERP responses to the non-drug-related arousing pictures differed from those to the neutral pictures, but did not differ from the responses to the drug-related pictures.

### Response to cue-induced craving questions

The responses to the question “Would you use cocaine (or marijuana) now if you could?” did not vary systematically during the ERP session within each group of participants. Overall, the cocaine-dependent participants reported that they would use the drug now if they could 52% of the time, while the cannabis-dependent participants said ‘yes’ 94% of the time. The responses to the question “What is your desire to use cocaine (or marijuana) right now?” displayed a similar difference between the groups, with mean scores of 26 for the cocaine-dependent participants and 63 for the cannabis-dependent participants on a scale of 0 to 100.

## Discussion

The technique of applying electrical stimulation to specific acupoints on the body to alter cocaine or cannabis use or cravings has not previously been evaluated. The results of the present pilot study suggest that TEAS does not cause robust changes in measures of drug use and daily drug cravings in cocaine- and cannabis-dependent participants who were not seeking treatment for their dependence. Neither drug use nor drug cravings were significantly decreased during 3.5 days of TEAS treatment. However, several interesting changes in other mood states were revealed, which may collectively play a role in drug abuse treatment strategies. Several ratings in the daily mood assessments were reduced in the cocaine- and cannabis-dependent participants, and the response to the drug-related pictures during the EEG assessment in the cocaine-dependent participants showed a nominal, but not statistically significant, reduction following TEAS treatment.

We analyzed the EEG/ERP P300 profiles for peak amplitude and latency to peak response with particular interest in the responses to drug-related cues for cocaine or cannabis. The altered peak voltage of the P300 EEG/ERP response in the cocaine-dependent participants may indicate a reduced response to drug-related cues. Despite a lack of statistical significance, the reduction in the P300 ERP amplitudes during the presentation of cocaine cues in the cocaine-dependent participants was consistent and remarkable, given the relatively small sample size and relatively short duration of TEAS treatment in the present study. Although subjective drug cravings and drug use in the cocaine-dependent participants was not reduced by TEAS treatment, examination of the subjective mood assessments in the cocaine-dependent participants revealed that TEAS treatment led to significant reductions in self-reports of anxiety, tension/agitation, and irritability, while concentration was improved. The present examination of the effects of TEAS treatment on cannabis cue reactivity and cravings assessed by the EEG/ERP P300 profiles for peak amplitude and latency to peak response in the cannabis-dependent participants did not reveal altered responses to cannabis-related cues. Similarly, subjective cannabis craving and self-reported use did not change as a result of TEAS treatment in the cannabis-dependent participants. TEAS treatment resulted in significant reductions in irritability and tension/agitation in the cannabis-dependent participants. These changes in the mood ratings may be of particular interest for cannabis dependence, because changes in these measures were previously identified as hallmarks of cannabis withdrawal during early abstinence [19]. Collectively, the findings indicate that TEAS treatment did not result in robust reductions in the EEG/ERP cue reactivity to drug-related pictures in either the cocaine- or cannabis-dependent participants. However, the observed reductions in negative mood measures resulting from TEAS treatment may have direct relationships with the modest reductions in drug cue reactivity observed for cocaine dependence. Improvements in these behavioral events may be useful in treatment settings, as previously noted by Meade *et al.*[20]. These authors previously demonstrated that, in addition to positive effects of TEAS as an adjunct treatment, there were marked improvements in mental health and self-reported physical health as a result of TEAS treatment compared with sham treatment during an outpatient follow-up period after inpatient opiate detoxification.

Several limitations of our study should be noted. The first is the duration of the assessment period for drug use, particularly for the cocaine-dependent participants. Significant alterations in cocaine use may not be observable during a 3-day assessment period. Since cocaine use is typically episodic and highly variable from day to day in many cocaine abusers [4,21], a true assessment of use may require several weeks. The brief drug-use assessment period would not appear to be a limitation for the cannabis-dependent subjects, because their use was regular and multiple times per day. Second, for practical reasons and equipment limitations, our treatments in this outpatient study were only administered twice a day, and only separated by 3 to 4 hours. Although changes in brain endogenous opioid levels have been observed from minutes to 1 hour after stimulation [1] and the effects on cocaine place preference can last for 24 hours [12], a proposal of three treatments per day separated by approximately 6 hours may be the most effective way to produce a maximal and sustained neurochemical alteration (Dr. Ji-Sheng Han, personal communication). Hence, there may be differential effects of TEAS treatment administered three times a day at 6 hours apart, compared with our TEAS treatment delivered only twice a day at 3 to 4 hours apart. Similarly, while a previous study of opioid dependence demonstrated positive effects of TEAS treatment using a similar number of TEAS treatment days [20], more robust findings from studies on opioids and positive findings for other drugs of abuse may result from TEAS periods with a greater number of treatment days. Third, the present study used alternating stimulation at 2 and 100 Hz. Although this method reliably increases endogenous opioids that activate mu, sigma, and kappa opioid receptors [13], antagonism of the effects of cocaine within the opioid system may primarily rely on the activation of kappa receptors. Ren *et al.*[12] showed that stimulation at 100 Hz, which acts primarily on kappa receptors, caused increases in the dynorphin levels and was effective for attenuating cocaine-induced conditioned place preference in rats. Finally, we chose to recruit individuals who were not seeking treatment for their dependence as a more rigorous test of the procedure. Additional studies will be needed to address these limitations, increase our sample size, and adjust the experimental protocols to explore the potential efficacy of TEAS on cocaine and cannabis use and cravings.

## Conclusion

Pharmacological and alternative medicine treatments for cocaine and cannabis abuse have not been developed to date. The present results suggest that the TEAS protocol used in this trial alone is not sufficient to significantly alter cocaine and cannabis use, drug cue reactivity, or drug cravings in cocaine- or cannabis-dependent individuals who are not seeking treatment for their dependence. However, it may be premature to state TEAS treatment is ineffective for use in cocaine or cannabis dependence, until TEAS treatment has been evaluated under a range of parameters, including longer treatment durations and different frequencies, and the contribution, if any, to reductions in negative moods can be assessed. Future assessments will need to evaluate the ability of TEAS to contribute to more comprehensive treatment regimens.

## Competing interests

The authors declare that they have no competing interests.

## Authors’ contributions

SEL designed the study as the principal investigator, and provided critical input for the manuscript preparation. DMP conducted the study, performed statistical analyses, and wrote the manuscript. ABR analyzed the drug use, drug cravings, and mood alterations. GHT conducted the EEG/ERP analyses and performed statistical analyses. RRM and SD conducted the daily sessions with the participants, performed initial data entry, and prepared graphs. DY-WL assisted the study design, analyses, and manuscript preparation. All authors read and approved the final manuscript.

## Supplementary Material

Additional file 1 Supplemental tables Click here for file
