# Supplementary material for: Effects of transcutaneous electric acupoint stimulation on drug use and responses to cue-induced craving: a pilot study
Source: Chin Med. 2012 Jun 10;7:14. doi: 10.1186/1749-8546-7-14 (PMC3412709; doi:10.1186/1749-8546-7-14)
Supplement: Additional file 1 — Supplemental tables [file 1749-8546-7-14-S1.doc]

Supplementary Tables

Table 1: Peak voltages by picture and type of treatment (mean ± SD) in µV.

|  | Neutral pictures | | Non-drug arousing pictures | | Drug-related pictures | |
| --- | --- | --- | --- | --- | --- | --- |
|  | Sham | TEAS | Sham | TEAS | Sham | TEAS |
| Cocaine-dependent participants |  |  |  |  |  |  |
| Oz | 9.8 ± 3.0 | 10.3 ± 3.5 | 10.9 ± 5.7 | 10.6 ± 7.9 | 15.2 ± 5.5 | 13.6 ± 4.7 |
| Pz | 8.4 ± 2.5 | 8.5 ± 2.8 | 13.0 ± 7.5 | 13.4 ± 7.1 | 17.1 ± 5.8 | 15.4 ± 5.9 |
| Cz | 5.9 ± 3.6 | 7.5 ± 3.9 | 10.1 ± 5.9 | 11.7 ± 5.9 | 11.4 ± 5.6 | 11.7 ± 6.1 |
| Fz | 3.6 ± 2.9 | 5.1 ± 2.7 | 7.2 ± 4.1 | 7.9 ± 3.4 | 8.0 ± 5.4 | 8.3 ± 5.3 |
| Cannabis-dependent participants |  |  |  |  |  |  |
| Oz | 16.2±15.3 | 14.3± 8.9 | 16.9±13.8 | 16.5±10.3 | 17.9±14.9 | 17.7±11.2 |
| Pz | 13.7±14.8 | 10.7±7.9 | 19.1±13.3 | 16.6±9.8 | 18.6±14.5 | 17.6±9.7 |
| Cz | 12.1±15.5 | 8.6±7.3 | 18.9±13.6 | 12.7±7.6 | 14.9±14.1 | 12.2±8.2 |
| Fz | 11.3±16.7 | 6.7±8.2 | 16.5±16.0 | 8.6±6.7 | 11.6±13.8 | 7.6±6.9 |

Table 2: Latencies to peak response by picture and type of treatment (mean ± SD) in ms

|  | Neutral pictures | | Non-drug arousing pictures | | Drug-related pictures | |
| --- | --- | --- | --- | --- | --- | --- |
|  | Sham | TEAS | Sham | TEAS | Sham | TEAS |
| Cocaine-dependent participants |  |  |  |  |  |  |
| Oz | 310.7 ±74.1 | 327.3 ±102.9 | 407.3 ±76.8 | 437.6 ±103.3 | 446.2 ±40.0 | 464.0 ±57.9 |
| Pz | 422.9 ±80.5 | 428.0 ±97.3 | 465.7 ±53.3 | 472.9 ±64.5 | 458.3 ±54.9 | 437.8 ±56.8 |
| Cz | 489.7 ±18.3 | 466.7 ±53.1 | 490.3 ±74.9 | 454.7 ±71.2 | 467.4 ±37.8 | 451.1 ±57.5 |
| Fz | 477.1 ±17.4 | 439.1 ±58.4 | 490.3 ±60.8 | 464.4 ±54.1 | 436.6 ±65.7 | 444.4 ±46.2 |
| Cannabis-dependent participants |  |  |  |  |  |  |
| Oz | 342.5 ±64.3 | 298.9 ±45.3 | 404.0 ±66.9 | 415.3 ±81.4 | 405.5 ±58.2 | 403.3 ±74.6 |
| Pz | 386.9 ±47.2 | 379.6 ±59.0 | 437.1 ±54.5 | 436.0 ±72.0 | 426.9 ±67.5 | 409.8 ±70.7 |
| Cz | 404.0 ±27.5 | 393.5 ±53.7 | 462.9 ±66.4 | 428.7 ±79.5 | 420.0 ±46.0 | 402.6 ±65.1 |
| Fz | 412.4 ±34.0 | 393.8 ±53.6 | 446.9 ±57.1 | 416.7 ±71.9 | 421.8 ±40.0 | 396.7 ±49.6 |
